# Supplementary figures and images for: Integrated Omics Analyses Identify Key Pathways Involved in Petiole Rigidity Formation in Sacred Lotus
Source: Int J Mol Sci. 2020 Jul 18;21(14):5087. doi: 10.3390/ijms21145087 (PMC7404260; doi:10.3390/ijms21145087)

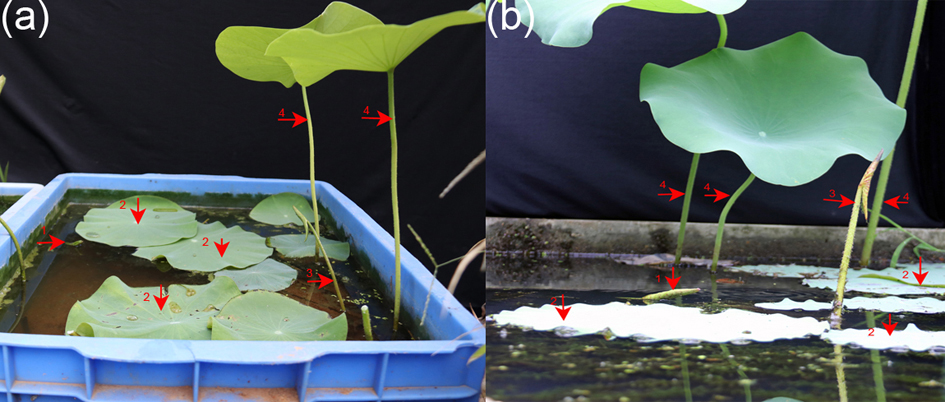

Supplement: Supplementary file 1 [file ijms-21-05087-s001.zip › Fig.S1.jpg]

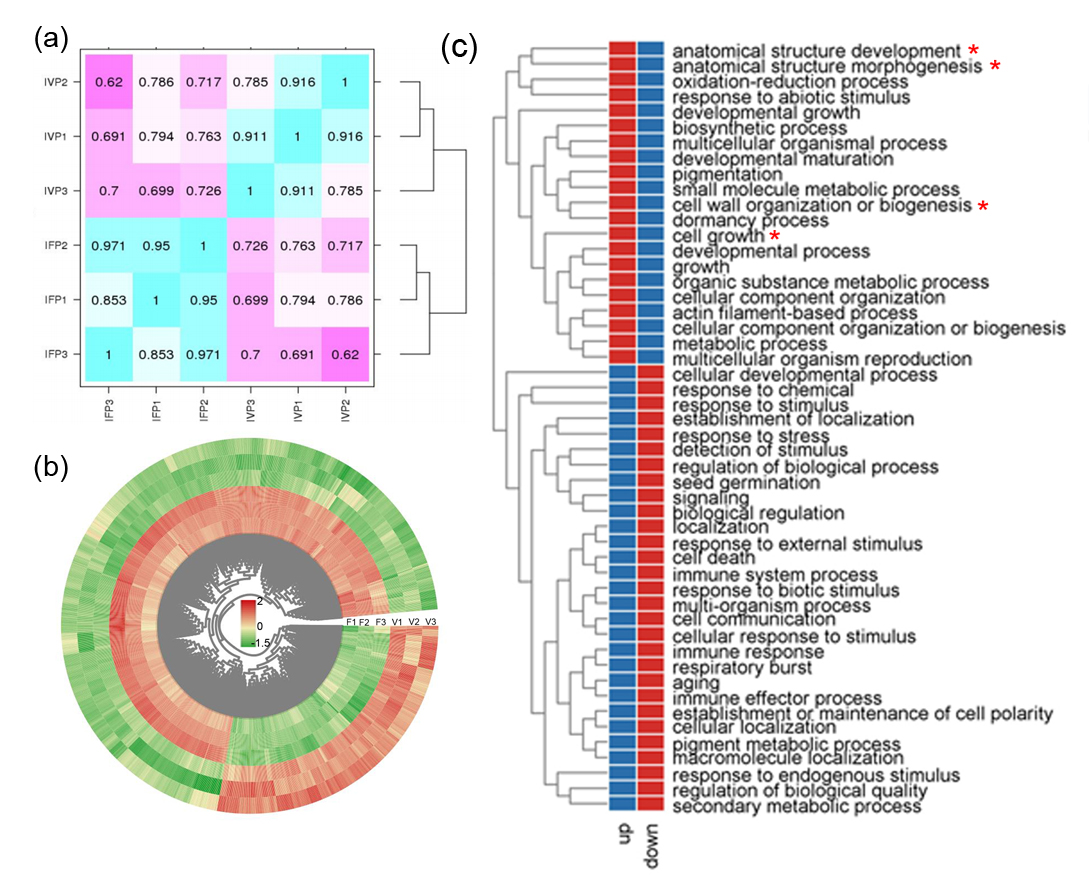

Supplement: Supplementary file 1 [file ijms-21-05087-s001.zip › Fig.S2.jpg]

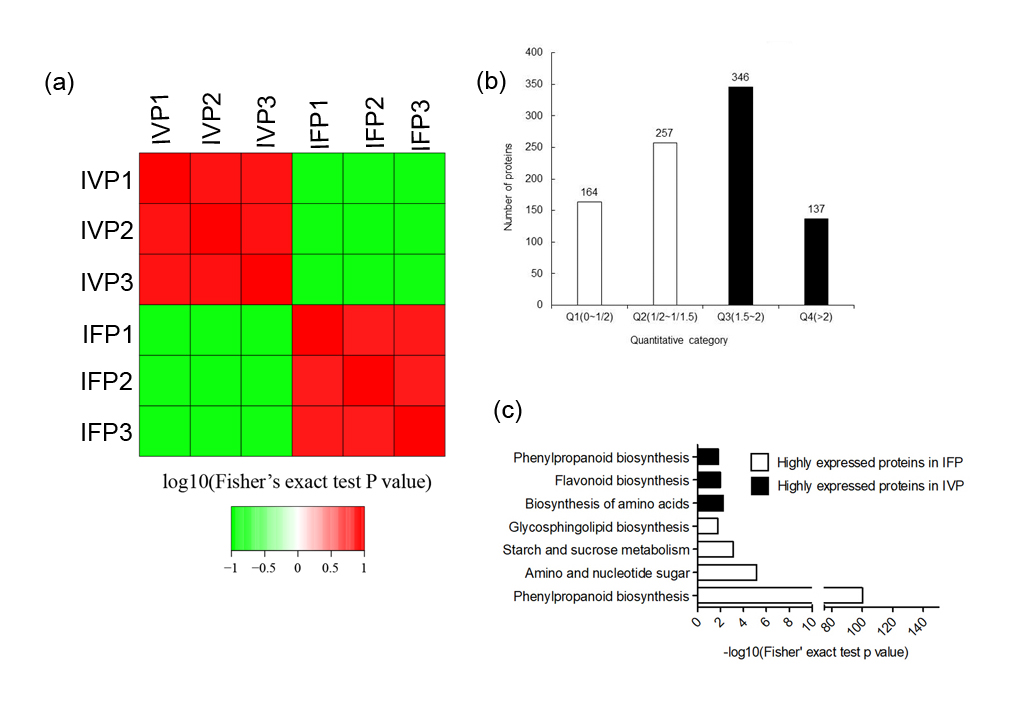

Supplement: Supplementary file 1 [file ijms-21-05087-s001.zip › Fig.S3.jpg]

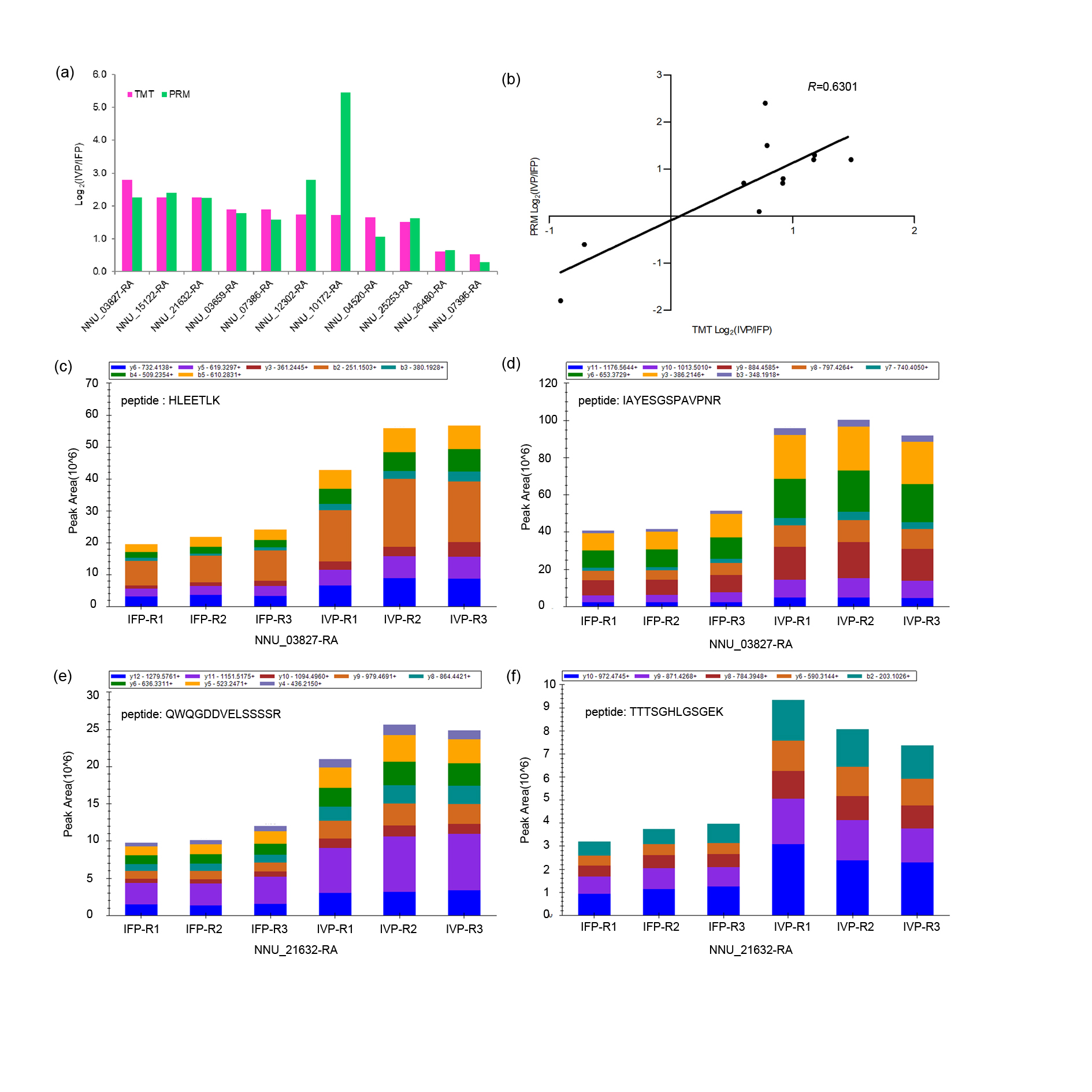

Supplement: Supplementary file 1 [file ijms-21-05087-s001.zip › Fig.S4.jpg]

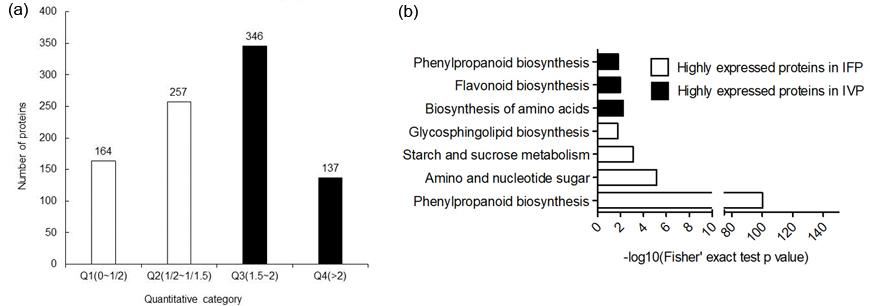

Supplement: Supplementary file 1 [file ijms-21-05087-s001.zip › Fig.S5.jpg]

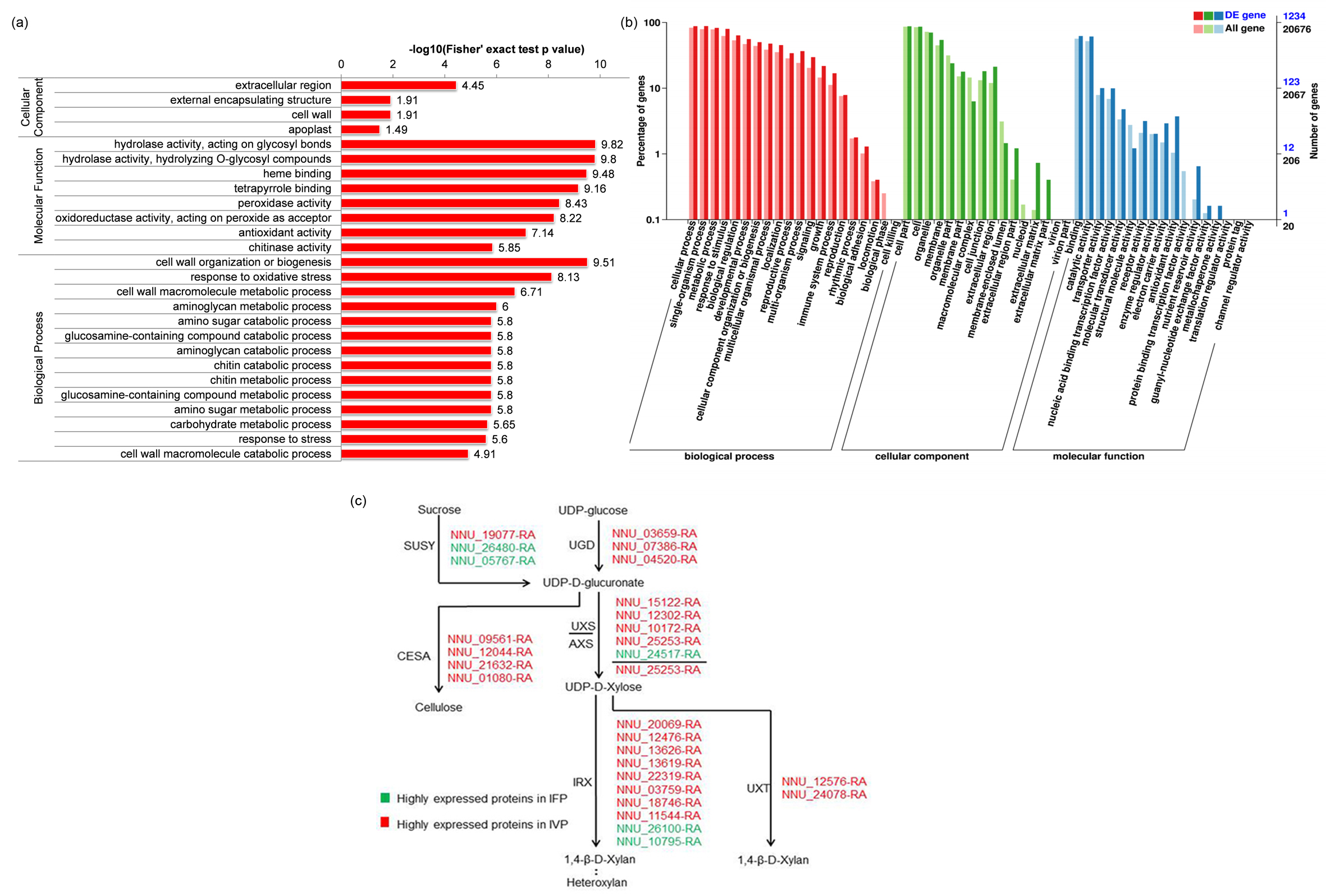

Supplement: Supplementary file 1 [file ijms-21-05087-s001.zip › Fig.S6.jpg]
